# Modeling Spike-Train Processing in the Cerebellum Granular Layer and Changes in Plasticity Reveal Single Neuron Effects in Neural Ensembles

**DOI:** 10.1155/2012/359529

**Published:** 2012-09-25

**Authors:** Chaitanya Medini, Bipin Nair, Egidio D'Angelo, Giovanni Naldi, Shyam Diwakar

**Affiliations:** ^1^Amrita School of Biotechnology, Amrita Vishwa Vidyapeetham (Amrita University), Amritapuri, Clappana, Kollam 690525, Kerala, India; ^2^Department of Physiology, University of Pavia, Via Forlanini 7, 21000 Pavia, Italy; ^3^Brain Connectivity Center, IRCCS C. Mondino, Via Mondino 2, 27100 Pavia, Italy; ^4^Department of Mathematics, University of Milan, Via Saldini 50, 27100 Milan, Italy

## Abstract

The cerebellum input stage has been known to perform combinatorial operations on input signals. In this paper, two types of mathematical models were used to reproduce the role of feed-forward inhibition and computation in the granular layer microcircuitry to investigate spike train processing. A simple spiking model and a biophysically-detailed model of the network were used to study signal recoding in the granular layer and to test observations like center-surround organization and time-window hypothesis in addition to effects of induced plasticity. Simulations suggest that simple neuron models may be used to abstract timing phenomenon in large networks, however detailed models were needed to reconstruct population coding via evoked local field potentials (LFP) and for simulating changes in synaptic plasticity. Our results also indicated that spatio-temporal code of the granular network is mainly controlled by the feed-forward inhibition from the Golgi cell synapses. Spike amplitude and total number of spikes were modulated by LTP and LTD. Reconstructing granular layer evoked-LFP suggests that granular layer propagates the nonlinearities of individual neurons. Simulations indicate that granular layer network operates a robust population code for a wide range of intervals, controlled by the Golgi cell inhibition and is regulated by the post-synaptic excitability.

## 1. Introduction

Decoding neural activity is the key to understand spatiotemporal patterns that the brain receives as sensory information regarding the world. Time-scale of operation is closely correlated to the activity of the neural circuit and decoding such activity reveals principles regarding the function. One of the main circuits in the cerebellum is the large input layer circuit formed of granule and Golgi cells. Spatiotemporal information is one of the unique functional characteristics observed in the cerebellar input layer network [1, 2]. Cerebellar granular layer forms the input stage of the cerebellum in which information coming from the peripheral and central systems converge through the mossy fibers. The granular layer has by far the smallest (~5 *μ*m) and the most numerous neurons (~10^11^) in humans. Understanding how the granular layer process information appears critical to understand the cerebellar function, since signals coming into upper cortical layers are provided by the granular layer. The granule cells form the largest neuronal population in the mammalian brain and regulate information transfer along the major afferent systems to the cerebellum. The granule layer receives excitatory input primarily from mossy fibers and inhibition via synapses from interneurons like Golgi cell. Mossy fiber input excites both the granule cell and inhibitory interneurons like the Golgi cell. The granule cell is a small neuron with 3–5 dendrites. Timing in the cerebellar granular layer plays a key role via passage-of-time representation (POT), learning or adaptation to movements [3, 4], modulation of information transfer to Purkinje cells (activation of granule cell subsets with respect to time). Knockout and lesion studies revealed that disruption of the granular layer leads to abnormal functioning of the cerebellar mossy fiber-granule cell relay [5], affects the learning-dependent timing of conditioning eyelid responses [6], loss of rapid spike processing in the cerebellum (results in ataxia) [7] which in turn affects the plasticity of granule-Purkinje cell synapses. Prion protein (PrP) knockout mice showed a large proportion of granule cells (~40%) with slow non-overshooting nonrepetitive action potential, slow EPSPs, and no inward rectification [5]. Likewise, FHF1-FHF4 mutants showed impaired granule cell excitability which prevented rapid burst transmission in the cerebellum [7].

The objective of the current paper is to study how excitation operates in the granular layer network [1, 2, 8–11]. The focus is also on understanding the modulatory role of inhibition [1, 2] in the granule cells [12] and underlying ensemble activity in terms of combinatorial operations on granular layer network [8].

In order to estimate spiking behaviour and to test reliability in modeling, we tried using detailed and simple models of neurons in our network models. The use of detailed multicompartmental models was focused towards reproducing the spatiotemporal dynamics of normal cerebellar activity. The detailed models allow reconstructing information including local field potentials [13], which were not seen while using less detailed models. We also used a spiking neuron model (modified from [14]) for reconstructing network activity in order to understand the contributions of individual spikes in the cerebellar cortex. The necessity of these spiking models was to retain the biologically plausibility of Hodgkin-Huxley-type dynamics, while maintaining the low computational cost. It was also an attempt to validate whether such simple spiking models also allow us to create computationally simpler yet large-scale models of cerebellum. Using properties of the granule cell [9] and Golgi neuron [15, 16], we developed simple spiking models [17] to represent the spiking behavior in a network. Estimates of spiking and reproducibility of spiking could be very useful for computationally efficient and large network models. Spike modulation due to the effect of feed-forward inhibition has been known to play an important role in time-windows hypothesis [18]. The simulations quantify how spikes pass through the granular layer network and the role of feed-forward inhibition in the neuronal microcircuit. This gives a new paradigm on the functional relevance of patterns in the cerebellar granular layer circuitry. The main objectives of the paper were also to reconstruct the center-surround excitation patterns and observe role of combined inhibition and excitation geometry in frequency-dependent transmission of spike information.

The objective was also to understand the effect of combinatorial operations on the granular layer network [8]. Combinatorial operations included combined excitation and inhibition which forms the spatiotemporal pattern in granular layer network *in vitro* and *in vivo*. Another objective behind the simulations was to understand information flow in granule neurons via burst-burst transmission and feed-forward inhibition [2]. Together they suggest the role of granule cells in expansion recoding and sparse activation via mossy fiber-granule cell synapses. The paper reports potential reconstruction of network activity in the form of center-surround structures [8], spike properties of underlying cells, and modulation of spikes due to changes induced by synaptic plasticity and due to inhibition. A special case of NMDA receptor blocking was simulated since GABA (*γ*-aminobutyric acid)-ergic inhibition is especially effective in controlling NMDA (N-methyl-D-aspartate) receptor-dependent depolarization in the granular layer [18]. Network simulations predict specific computational roles of granule cells in processing bursts and overall spike processing in the cerebellar granular layer.

Understanding population code via comparisons of spatiotemporal properties of simulated neural activity and with experimental measurements using multielectrode recordings [18] is useful to identify how information encoding happens in microcircuits. Therefore, evoked local field potential (LFP) responses from granular layer *in vitro* [19] were reconstructed computationally [20]. The main intention of local field reconstruction on the network *in vitro* was to study the role of inhibition in generating the N_2b_ wave [4, 20]. The paper also investigates the combined role of excitation-inhibition affecting the granular layer clusters.

## 2. Methods

The study carried out in this paper involved the use of computational models of neurons based on experimental data from p17–23 Wistar rat cerebellum [9]. Mathematical neuron models of granule cell (GrC) [9, 19] and Golgi cell model (GoC) [15, 16] were used in this network study. Modeling reliability for spiking models was based on the extensive characterization of membrane currents and the compact electrotonic structure of cerebellar granule cells [9, 19]. The models used AMPA (2-amino-3-(5-methyl-3-oxo-1,2-oxazol-4-yl) propanoic acid) and NMDA receptor components as excitatory mossy fiber (MF)-GrC synapses and GABAergic synapses for the GoC-GrC relay [1, 15, 19, 21]. On an average, each granule cell receives excitatory connections from 4-5 mossy fibers [9].

### 2.1. Simple Neuronal Models

The objective of using simple models in the study was to understand how spatiotemporal patterns integrated over time to produce responses that are selective to specific patterns and to reconstruct the representation of spiking behavior in networks especially to study how inhibition affects intrinsic electroresponsiveness. We also wanted to see how the high variability in MF spike trains affects firing behavior. A simple spiking model [14] was used to study the neuronal spiking activity. A good model should be feasible with Hodgkin-Huxley dynamics and be computationally efficient [14] to reproduce the firing behavior of biorealistic model. The simple spiking model [14] of neuron primarily used two equations one regulating the membrane potential (*V*) and the other regulating adaptation current (*w*)

(1) $$\begin{array}{c} C\frac{dV}{dt}=-g_{L}\left(V-E_{L}\right)+g_{L}\Delta T\;\exp\left(\frac{V-V_{T}}{\Delta T}\right)-w+I \end{array}$$

(2) $$\begin{array}{c} t_{w}\frac{dw}{dt}=a\left(V-E_{L}\right)-w, \end{array}$$

where *C* is the membrane capacitance, g_L_ is the leak conductance, *V*_*T*_ is the voltage threshold, Δ*T* is the slope factor which quantifies the sharpness of the spikes, *w* is an adaptation current, and *I* is the injected current. The membrane time constant is

(3) $$\begin{array}{c} t_{m}=\frac{C}{g_{L}}. \end{array}$$

The reset values were activated when the membrane potential reached the desired peak voltage:

(4) $$\begin{array}{c} \text{if}\;\left(V>30\;\text{mV}\right),\text{then}\\ V\;=\;V_{r}\\ w\;=\;w\;+\;b. \end{array}$$

The change in parameters (as shown in (4)) allowed the simple spiking model to replicate amplitude of granule cell firing and Golgi cell firing (see Table 1). Capacitance values of Golgi and granule cells were modelled to simulate similar frequency and firing patterns matching experimentally observed data, as observed in biophysical models [9, 15].

While replicating the basic firing behavior, the Adaptive Exponential Integrate-and-fire (AdEx, see [14]) granule cell model was tested with various combinations of synaptic connections. AMPA [17, 22, 23] synaptic kinetics was used as the excitatory synaptic dynamics and GABA [17, 24, 25] as the inhibitory synaptic kinetics as observed via experiments [19]:

(5) $$\begin{array}{c} g_{\text{AMPA}}=g_{\text{AMPA},\max}*\;e^{-t/18}*\frac{1-e^{-t/2.2}}{0.68}\\ I_{\text{AMPA}}=\left(V_{m}-\;0.0\right)*\;g_{\text{AMPA}}. \end{array}$$

Likewise, GABA synaptic kinetics was modeled using the GABA-A equation [17]:

(6) $$\begin{array}{c} g_{\text{GABA}}=\;g_{\text{GABA},\max}*\;e^{-t/25}*\frac{\left(1-e^{-t/1.0}\right)}{0.84}\\ I_{\text{GABA}}=\left(V_{m}+\;75\right)*\;g_{\text{GABA}}. \end{array}$$

The maximal conductance of AMPA and GABA was adjusted to suit the observed biophysical firing pattern (see Table 2). For simulating the case without inhibition, maximal conductance value of GABA synapses is set to zero (see Table 2). To model inhibition, varying values of maximal conductance for different number of inhibitory synapses were used. With these two receptor kinetics, we were able to match the number of spikes and amplitude to that of biophysical model. The models also reproduced a close correlation to the biophysical model while varying the intrinsic excitability and release probability (data not shown). By changing conductance and dynamic parameters, we simulated LTP and LTD, matching to experimental and computational values [26].

To understand network dynamics in terms of firing and temporal processing, we used a simple spiking network with 1680 AdEx granule cell models and 1 AdEx Golgi cell model. The properties of the network model were matched (see Tables 1 and 2 and [9, 15, 16, 27, 28]) to experimental data.

### 2.2. Multicompartmental Models

A detailed multicompartmental GrC model [19] was used, and simulations were performed by varying the excitatory (*E*) and inhibitory (*I*) synaptic inputs. The model of the granule cell was based on multicompartmental cable theory and included soma, axon, hillock and dendritic compartments. The model consisted of 52 active compartments connected to each other via the 3/2-power law [29]. For each of the compartments, membrane voltage *V*_*m*_ had to be estimated separately:

(7) dVmdt=1τm(V−∑igi(V−Vi)+∑syngsyn(V−Vsyn)+∑brgbr(V−Vbr)gtot),

where *g* is the conductance corresponding to *i* (ion channel), syn (synaptic dynamics), br (neighboring attached branch), and tot (total). Here, *τ*_*m*_ = *R*_*m*_ *C*_*m*_ which is the time constant of oscillation of the membrane based on its membrance resistance, *R*_*m*_ and membrane capacitance, *C*_*m*_. The calcium current in the model was included as

(8) $$\begin{array}{c} \frac{d\left[\text{Ca}\right]}{dt}=-\frac{I_{\text{Ca}}}{\left(2F\cdot A\cdot d\right)}-\left(\beta_{\text{Ca}}\left(\left[\text{Ca}\right]-{\left[\text{Ca}\right]}_{O}\right)\right), \end{array}$$

where *d* is the depth of a shell adjacent to the cell surface of area *A*, *β*_Ca_ determines the loss of calcium ions from the shell approximating the effect of fluxes, ionic pumps, diffusion, and buffers, [Ca]_*O*_ is resting [Ca], and *F* is the Faraday's constant. [Ca] is the calcium channel dynamics as reported in [19].

 The model GrC has 1–4 excitatory (one for each dendrite) and 0 (no inhibition)–4 inhibitory connections (one for each dendrite) [19]. The detailed explanations of ionic channel dynamics, compartmental localization of ion channels and electronic structure of this granule neuron model are described elsewhere [9, 19, 21]. Since granule cell is one of the rarest neurons where the ionic channel densities can be accurately determined using whole-cell patch clamp, the ion channel dynamics that was modeled previously [7, 9, 21] is not repeated here. Also, excitatory and inhibitory synaptic inputs to the dendrites were located in dendritic tips although in neighboring dendritic compartments. Presynaptic dynamics for the MF-GrC was modeled separately as in [19, 21] due to components such as facilitation and depression. Excitatory postsynaptic mechanisms were shown as AMPA and NMDA postsynaptic receptor components as seen in granule neurons. AMPA receptor dynamics was modeled using a three-state scheme and a 2D diffusion model, whereas the NMDA receptors used Boltzmann equation as seen in [30]. Both the excitatory presynaptic and excitatory postsynaptic mechanisms are described in detail elsewhere [21]. The GoC-GrC inhibitory synapse model was based on the following presynaptic dynamics: release probability = 0.35, *τ*_REC_ = 36 ms, *τ*_facil_ = 58.5 ms, and *τ*_*I*_ = 0.1 ms, respectively and as described in [31]. Effects of blocking inhibition by adding gabazine were also simulated by setting GABAergic conductance in inhibitory fibers to zero.

 The Golgi neuron was adapted from [15, 16]. All simulations were performed with NEURON environment [32] running on HP Blade C3000 node. Timing and initial time-window modulations are mainly affected by the role of feed-forward inhibition as it happens with only a slight delay from the mossy-fiber input and hence the role of feed-back inhibition was not simulated.

### 2.3. Granular Layer Network

**Figure 1:**
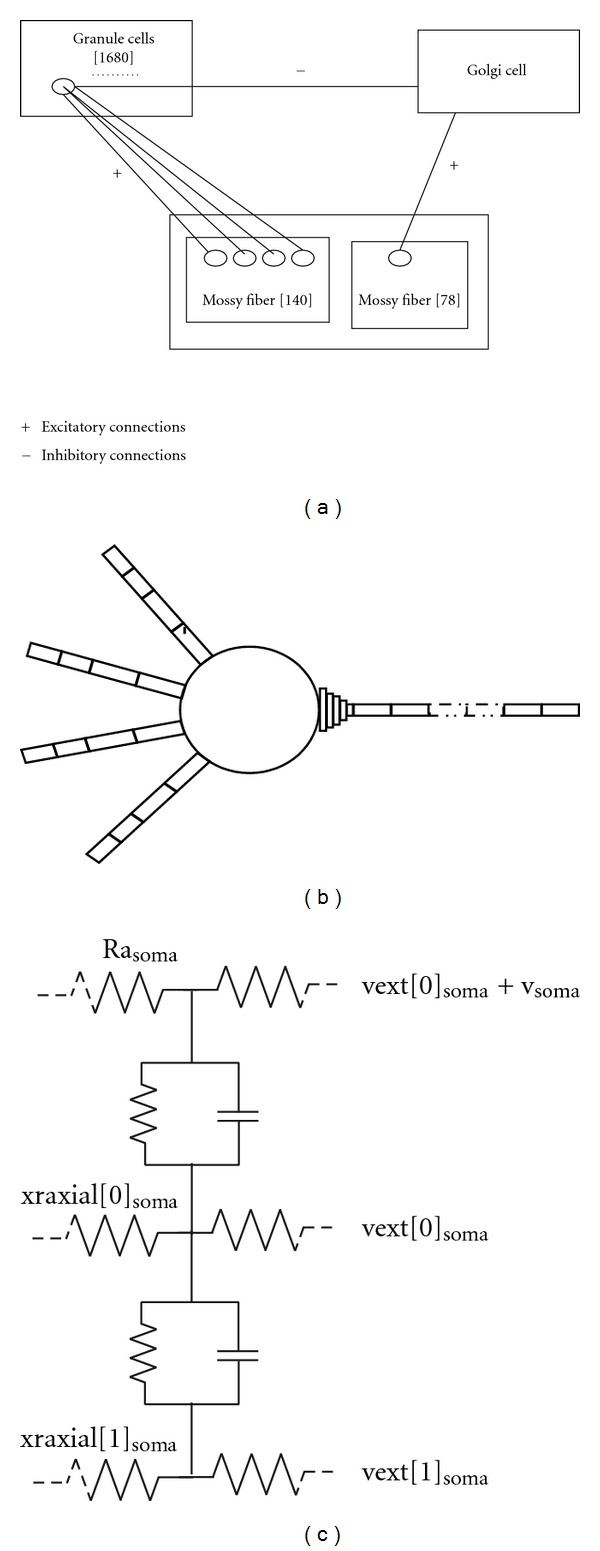
Granular layer network topology. (a) Network topology map. Granule cells (GrCs) receive 1–4 excitatory inputs from mossy fibers. GrC receive 0 (no inhibition)–4 inhibitory inputs from GoC via the GABAergic synapses, one per granule neuron dendrite. The ratio is about 4000 : 1 [1]. Granular layer processing is fast and usually output spikes are seen in millisecond time intervals. (b) Detailed granule neuron model adapted from [19]. (c) Extracellular mechanism to study extracellular current flow in compartmental models. This mechanism was used to model LFP (see Section 2.6).

Granular layer spiking network model consisted of 140 homogenous mossy fibers (MF) rosettes, 1680 granule cells (GrC), and 1 Golgi cell (GoC). In this network, about 48 GrC receive 1 excitatory input from the same mossy fiber, and each granule cell receives four excitatory connections from four different mossy fibers. Along with these excitatory inputs given to GrC, mossy fibers also provide excitatory input to GoC whose ratio was set in this model to about 78 : 1 (see [15, 16], each dendrite had 26 synapses in the GoC model and assuming a total of 3 dendrites, we approximated to 78 synapses) providing an overall glomeruli connectivity pattern [1]. The network topology is illustrated in Figure 1(a).

Modeling responses in brain slices *in vitro* were simulated by giving single spike as input via mossy fiber (MF) terminals. Anaesthetized rat brain recordings *in vivo* showed bursts as inputs through mossy fibers [33]. Therefore, *in vivo* inputs to GrC were simulated as bursts of (5 or 9) spikes via the MF input.

### 2.4. Center-Surround “Spot” Pattern

Stimulating mossy fibers with an electrode at a particular point activates granule cells in the network in a center-surround activation pattern [8]. Within a “spot,” cells which are in close proximity to the electrode will receive high excitation and the periphery layer cells receive less excitation. In the network model, we simulated the center-surround pattern (see Figure 4A in [8]) defined as a “spot,” showing decreasing strengths of excitation spreading from the center to the periphery. In each spot, 48 cells in the center received 4 mossy fiber (MF) inputs, 144 cells received 3 MF inputs, 48 cells had 2 MF inputs, and 144 cells received 1 MF input.

### 2.5. Simulating LTP/LTD

The granule cell model was modeled based on data from Wistar rats [9, 19]. By modifying intrinsic excitability and release probability [34, 35], we simulated plasticity in the GrC. Intrinsic excitability was modified by changing ionic current channel gating dynamics. On-off gating characteristics of sodium channel were altered to modify sodium activation and inactivation parameters [13, 26] for higher and lower intrinsic excitability. Three cases were studied where the intrinsic excitability of the GrC is low (low intrinsic excitability), normal (control), and high (high intrinsic excitability). The release probability (*U*) of MFs varied from 0.1 to 0.4 for cells with low intrinsic excitability, and from 0.5 to 0.8 for cells with high intrinsic excitability while the control value remained as 0.416 in simulations for normal cells [36].

### 2.6. LFP Reconstruction

The extracellular potential of a single granule neuron (see Figure 1(b)) was estimated using NEURON [32] extracellular mechanism. The mechanism adds two RC compartments (see Figure 1(c)). To understand population code, we reconstructed network evoked LFP response using Laplace equation (see (1)):

(9) $$\begin{array}{c} \nabla^{2}\emptyset =0, \end{array}$$

where *∅* is extracellular potential, at boundary condition (1/*ρ*)*∅* = *J*_*m*_ · *J*_*m*_ is the transmembrane current density and *ρ* is the extracellular resistivity. Each cell generated an extracellular response corresponding to the activation pattern elicited by the mossy fibers. With the granular layer network, an electrode was assumed to be placed at the center. Temporal and spatial delays due to distance from electrode were assumed to be 0–3 ms [37]. The electrode could measure cells that generated extracellular currents that came with a delay of 0–3 ms (see [20]). Methodology for modeling the latencies used has been detailed elsewhere [13]. Considering the extracellular activity from each granule cell in the region of interest (number of cells = 700, assuming measurements from a tungsten electrode [18]), we reconstructed evoked LFP response using (10) and (11). Equation (10) adds the delay by padding zeros to linearly time-shift the signal. Equation (11) denotes the process of summing all shifted extracellular signals for all cells linearly. Total signal obtained is the desired evoked LFP:

(10) $$\begin{array}{c} \emptyset_{\text{shifted},i}\left(t\right)=\;\emptyset_{i}\left(t-t^{\prime }\right) \end{array}$$

(11) $$\begin{array}{c} \emptyset_{\text{evoked}\;\;\text{LFP}}\left(t\right)=\;\sum_{i=0}^{n}\emptyset_{\text{shifted},i}\left(t\right), \end{array}$$

where *∅*_*i*_(*t*) is the extracellular potential of *i*th cell in the neuronal population within the region of interest. *∅*_shifted,*i*_(*t*) represents the extracellular potential shifted by time delay (0–3 ms) (see [33]). Equations (10) and (11) were calculated separately. The detailed methodology for reconstructing evoked local field potential (LFP) has been described elsewhere [13].

## 3. Results

We were able to construct two models of granular layer network microcircuit: one using computationally efficient but physiologically limited spiking neurons and other using biophysically detailed multicompartmental neurons and reproduced activation patterns, burst-burst transmission, role of inhibition, and combinatorial coding.

### 3.1. Time-Windowing Depends on the Feed-Forward Inhibition-Implications from Simple Spiking Network Model

The objective of using a simple spiking model was to understand input-output relationships in terms of firing dynamics in the cerebellar granular layer.

**Figure 2:**
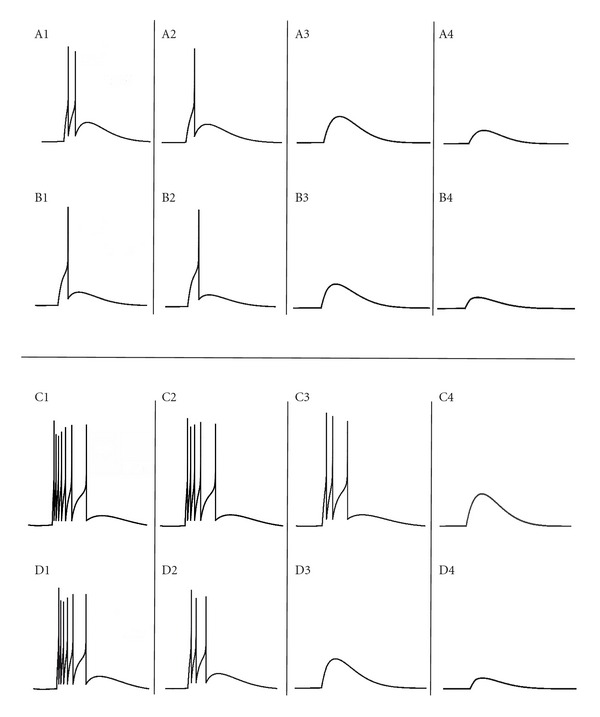
Firing patterns observed with AdEx model. (A) and (B) shows the *in vitro* behavior receiving 1 spike through the MF synapses. *In vivo* behavior (burst-burst transmission) is simulated (C) and (D) via bursts through the MF synapses. (A) and (C) show traces with no inhibition while (B) and (D) show traces with inhibition. Responses from left to right indicate input activation patterns from 4 MF excitations to 1 MF excitation. The AdEx model faithfully reproduced granule cell spiking behavior *in vitro* and *in vivo* [9, 19, 28].

The simulated single granule neuron responses were modeled based on granule neuron electroresponsiveness [9, 28]. Both *in vitro* (see Figure 2(A)) like behavior with single spike through mossy fibers and *in vivo* (See Figure 2(C)) like response with burst inputs through mossy fibers were simulated. The responses matched experimental data [9, 19, 28]. The role of feed-forward inhibition [31, 38–40] was also modeled. With inhibition, the granule neuron model showed suppression (see Figure 2(B1)) of spike doublet (see Figure 2(A1)). Synaptic inhibition, because of its delayed activation, controlled generation of the second spike in the doublet [18, 41]. In the *in vivo* case, the number of spikes (see Figure 2(C)) was reduced due to inhibition (see Figure 2(D)).

Using the spiking granule neuron model, the 1680 granule cell network was reconstructed. The synaptic input in the mossy fibers were reproduced using either a single pulse to mimic electrical stimulation for *in vitro* simulations or short high-frequency trains mimicking punctuate sensory stimulation for *in vivo* simulations The network model showed 720 spikes (without inhibition) and reproduced the synaptic activation of the granular layer.

**Figure 3:**
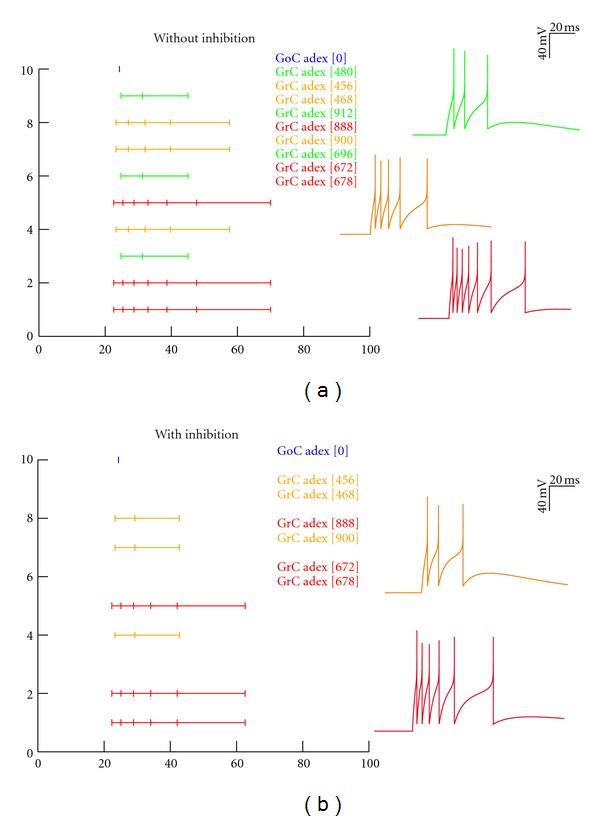
Spike raster plots for the network *in vivo* with AdEx models. Network model using AdEx [14] neurons reproduce the spike raster for *in vivo* firing dynamics. A short burst of 5 spikes at 500 Hz was given as inputs through the MF. Feed-forward inhibition affected the network by reducing number of spikes (b). Network without inhibition (a) shows 1–7 spikes and simulates the role of Gabazine that blocks GABAergic synapse.

The spiking neuron network model was activated with a center-surround activation pattern [8], and the raster of spikes were observed in individual cells. Among 1680 cells, 144 cells with 4 MF active, 432 cells with 3 MF, 144 with 2 MF, and 432 with 1 MF active. The configuration was based on Voltage-Sensitive Dye (VSD) imaging [8] and results matched our previous findings [42]. With LTP and LTD, the numbers of spikes in the network change significantly (see Table 3). The spiking neuron simulations supported a burst-burst transmission modality (see Figure 3) in which high-frequency spike trains are more reliably transmitted.

The time-windowing [1] depended on the feed-forward inhibitory loop, regulated by the Golgi synapses impinging on the granule neurons. In this model, inhibition was modeled with a delay of 4 ms to account the MF-GoC-GrC circuit. As expected, the feed-forward inhibition reduced the number of spikes from 720 to 576 (see Table 3). The spike raster in the simulations (see Figure 3) showed selective inhibition of granule firing due to blocking of the second input as reported in [1, 2, 18]. The increase in number of spikes *in vivo* in the network supports the frequency-modulated transition from LTP to LTD [43]. The model also was computationally efficient in comparison to the biophysical model (see Section 3.2) and took 3 s for a 100 ms simulation.

### 3.2. Spike-Burst Generation and Bidirectional Plasticity

Although simple spiking models allow reconstruction of frequency and amplitude information in terms of firing of constituent cells, role of plasticity and selective pharmacological effects in population code could not be studied. As reported in Section 2.2, we used detailed multicompartmental models [15, 16, 19] to generate a 1680 granule cell network. A detailed model allows to focus on understanding how specific temporal dynamics and the geometry of connections will eventually determine the circuit output, as indicated by the evident anomalies in network functioning and behavior caused by single-gene mutations altering the physiology of single molecules or neurons [2]. The simulations also attempt to understand and indicate certain functional theories of feed-forward inhibition, sparse recoding via, spikes and long-term plasticity.

LTP in granule cells [21] comprises of variation in release probability and intrinsic excitability. The network model was *modified* with higher intrinsic excitability observed by changes to sodium channel properties and release probabilities of MF synapses, thereby simulating granule cell LTP. LTD [43] was also simulated by combining lower intrinsic excitability and low release probability. With varying amounts of excitation, cells generated different number of spikes. In the simulations, granule cells generated repetitive nonadapting spike discharge in response to a continuous stimulus [27, 28, 45]. Simulations on single granule neurons *in vitro* suggested that granule neurons allowed spike burst generation and resonance in a low-frequency band (between 4 and 10 Hz) [9, 46]. High-frequency bursting [44, 47–49] was simulated to characterize network properties of granule cells *in vivo* [26, 33].

### 3.3. Network Excitability Changes with Varying Excitatory Release Probabilities

During events such as in epileptic seizures, heterogeneous spiking activity is noticed [50]. To understand the nature of spiking, we simulated the role of excitation via mossy fiber. We simulated single spikes (low frequency) and bursts (high frequency) so as to understand spiking behavior *in vitro* and *in vivo.*

In the case of simulating *in vitro *behavior (see Section 2.3) in granular layer network, with release probability 0.416 (control) the cells with normal intrinsic excitability receiving 4 excitatory inputs produced spike doublet and cells receiving 3 excitatory inputs produced single spikes (see granule neuron electroresponsiveness in [19]). Cells receiving 2 excitatory and 1 excitatory inputs did not produce any spikes [19].

Decreasing MF synapse release probability from 0.3 to 0.1, many granule cells in the network did not generate spikes. With increased release probability of MF synapses from 0.42 to 0.6, there was an increase in number of spikes (the number of spiking cells increased from 192 to 432) with no significant change in first spike latency and spike amplitude [36].

With the higher release probabilities like 0.7, 0.8 of MF synapses, the number of spikes saturated and the number of spiking cells remained the same (as seen in 0.6 release probability of MF synapses). Varying synaptic release probabilities, it was possible to generate selective responses.

Increasing intrinsic excitability from normal to higher excitability by modifying sodium gating properties (see Section 2.5) showed a significant increase in the spike amplitude (~6%) for all spiking cells, and an increased number of spikes was observed only for the cells with higher release probability and number of active MF synapses. This change corresponded to long-term potentiation in granule neurons [21] confirming the mechanisms role in spiking and bursting. The number of spiking cells varied from 192 to 432 in the network of 1680 granule cells.

With *in vivo* inputs, the number of cells showed a greater sensitivity with LTP (see Table 3) and the number of nonspiking cells decreased. LTD showed decrease in firing-nonfiring [43] cell ratio. The ratio of firing cells did not change with the length of the burst (see Table 4).

### 3.4. Inhibition and Spike-Count Modulation

Golgi cells can control both the temporal dynamics and the spatial distribution of information transmitted through the cerebellar granular layer network [43]. The strength of the inhibition depends on the number of inhibitory connections and synaptic release probability. The dynamics of the granule cells-Golgi cell circuit were explained by the simultaneous activation of both neurons through the mossy fibers, followed by activation of the feed-forward and feed-back inhibitory loops [18, 51]. The granule and Golgi cell received excitatory inputs from mossy fiber (MF) at the same time. There are two basic patterns of mossy fiber activity that can activate the Golgi cells, namely, protracted frequency-modulated discharges and short high-frequency bursts [48, 52]. The inhibitory input from Golgi cell reaches the granule cell with a loop delay of approximately 4 ms [53] compared to the mossy fiber input through GABAergic synapses [2]. The inhibition-based time-windowing in granule cells allow one or more spikes and is seemingly regulated by varying inhibitory inputs.

**Figure 4:**
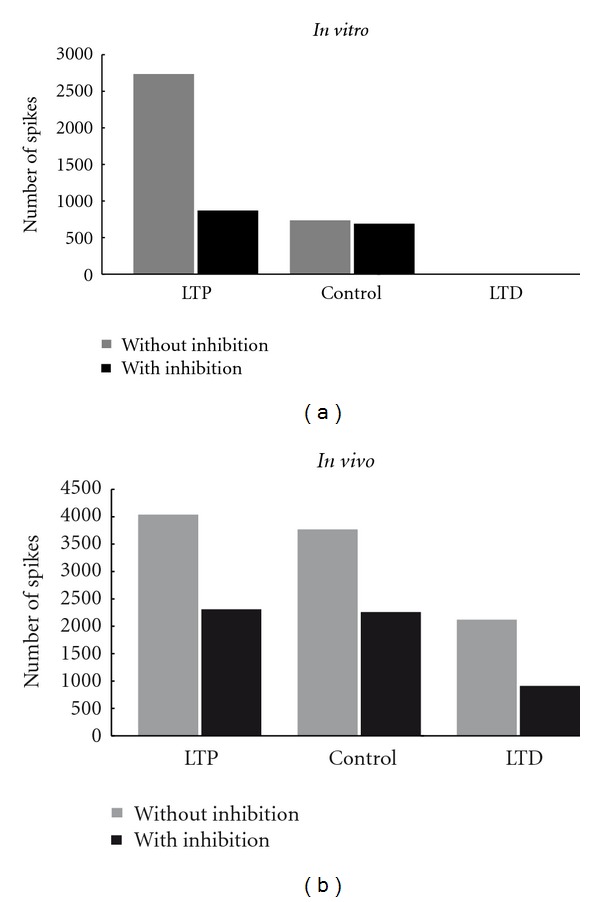
Histogram showing the effect of LTP and LTD on spiking in the network built with detailed biophysical models. Under *in vitro* (a) like spike input via MF, the number of spikes changed from 720 to 2736 (gray bars). Control refers to the excitatory release probability, *U*, which was set to 0.416. The presence of inhibition showed a sharp modulation, and the number of spikes seen in the network was 576 and during LTP it increased to 874 (black bars). LTD showed no spikes. Under *in vivo *(b), the change in number of spikes from control to LTP was 3600 to 4032 and during LTD was 2016 (gray bars). With inhibition (black bar), the number of spikes in the 1680 cell network decreased to 2304 (LTP), 2160 (control), and 864 (LTD).

Golgi cells converging through lateral connections onto some granule cell subsets could generate combined inhibition [2, 8]. The impact of the inhibition on granular layer circuitry differs with respect to two different properties: amount of inhibitory connections and the GABAergic release probability. The variation in the number of spikes with and without inhibition was significant in both cases *in vitro *(see Figure 4(a)) and *in vivo *(see Figure 4(b)). As expected, LTP showed increased number of spikes compared to control, while LTD showed reduced number of spikes. *In vitro* LTD suppressed spikes (see Figure 4(a)).

During simulations as seen *in vitro* (see Figure 4(a)), increased inhibition regulated the spike count rather than affecting the number of spiking cells. Short burst through MF produced ~7 spikes in single neurons, but inhibition showed a sharp modulation by regulating the time-window. A long burst produced slower modulation of spikes in the network.

**Figure 5:**
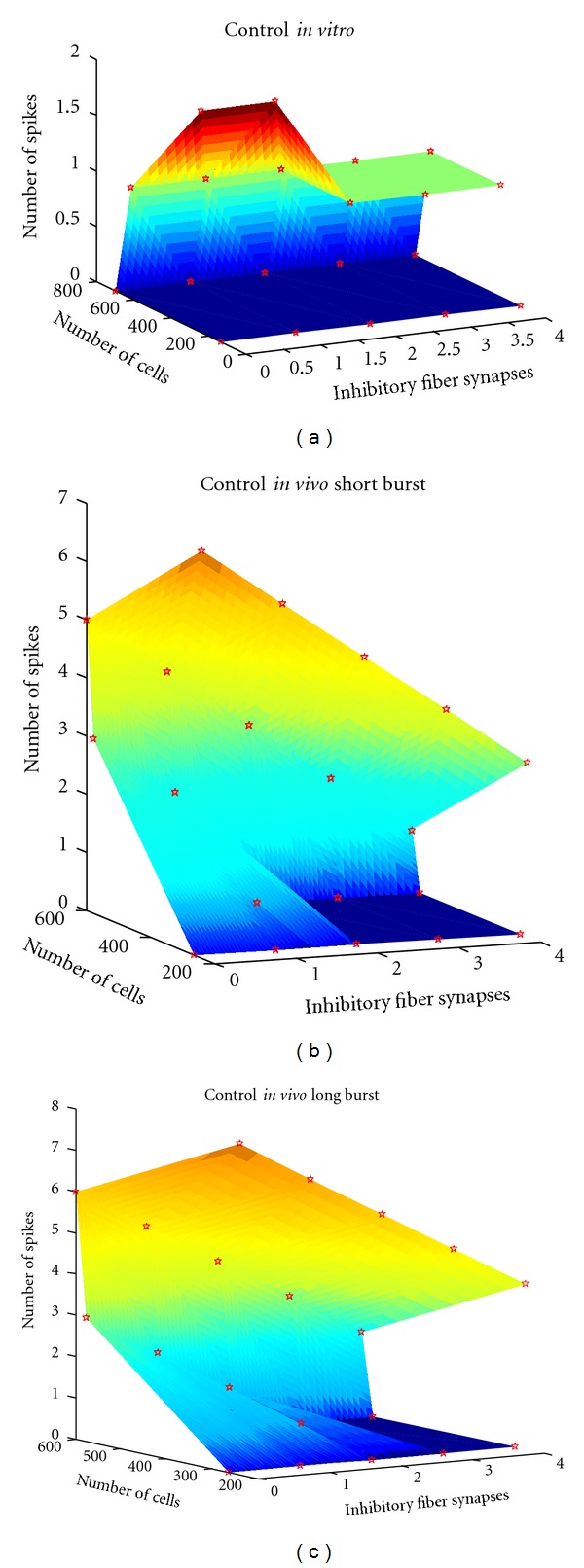
Effect of inhibition on variation in number of spikes and spiking cells. With varying inhibition, the number of spiking cells and total number of spikes varied. With 1 spike via MF as input ((a), *in vitro* behaviour), the total number of spiking cells varied from 200–600 cells and 1-2 spikes modulated by the inhibitory inputs (*x*-axis). Tactile stimulation induced two types of bursts *in vivo *[33]. (b) shows the number of spiking cells *in vivo* (as short burst of 5 spikes at 500 Hz via MF) with respect to changes in number of spikes as inhibitory inputs (*x*-axis) were changed. Variation in the number of spiking cells and number of spikes is shown. With a longer burst (9 spikes at 500 Hz via MF) *in vivo* (c), there were more spikes and inhibition did not cause a sharp change in number of spikes or spiking cells in the network (see also Table 5). The number of active cells can be observed also by looking at the increase of the number of “silent” cells. The granule cells favour a better role as signal-to-noise enhancers in the network [44] and facilitate burst-burst transmission.

The increase in inhibitory connections (see Table 5 and Figure 5) to granule cells in the underlying network model decreased number of spikes (see spike count in Figure 5, control refers to release probability being set at normal condition, *U* = 0.416), spike amplitude (if the spike rises after the 4 ms time-window when inhibitory inputs reaches the granule cell) and decreased spike latency.

Changing inhibitory (GABAergic) synapse release probability (*U*_inh_), spike amplitude, and first spike latency were affected [42]. Spike amplitude decreases whereas spike latency remains unchanged, when *U*_inh_ varied [42].

The increase of inhibitory input increases the number of silent cells (visible by the blue plateau in Figure 4), therefore reducing the number of active cells. The simulations indicate that the response of those granule cells that are intensely activated will favor with the generation of a burst, regulated mainly by feed-forward Golgi cell inhibition (see Figure 5 and [48]).

### 3.5. Center-Surround Excitation in Populations of Granule Cells

**Figure 6:**
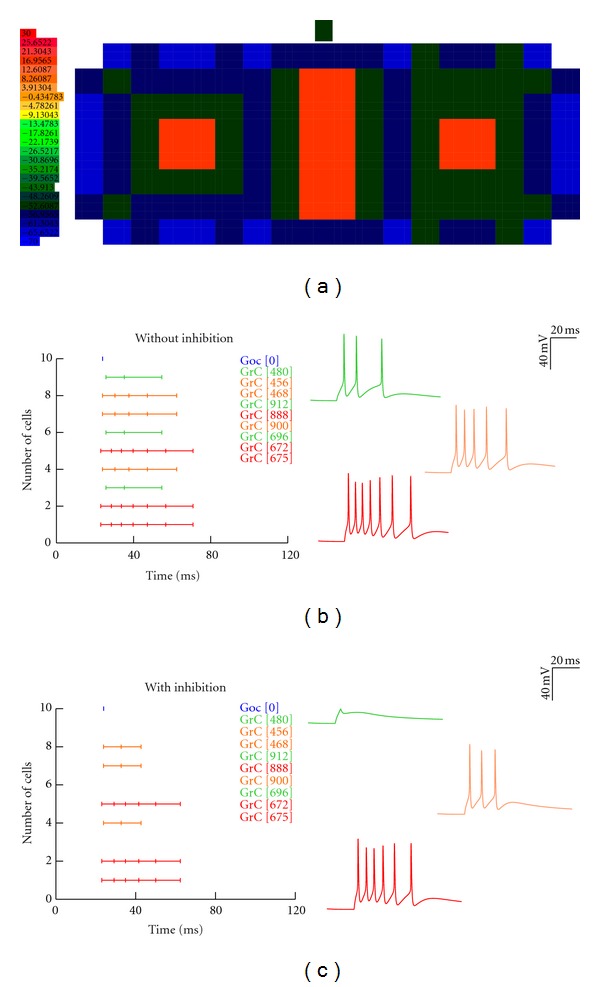
Center-surround “spot” activation. Varying levels of synaptic excitation in the spot (a) as mossy fiber inputs to granule cells in the network were reconstructed. Three spots of which each spot had 384 granule cells (see Section 2.4), and the excitation potential was indicated by the colormap. In the model configuration (a), the center of the spot receives stronger excitatory inputs and the consecutive peripheral neurons receive weaker excitatory input, thereby expressing a center-surround configuration. Compared to the surround, the center detects burst on a broader band and emits bursts with shorter lag, higher frequency, and longer duration [1]. Network model using detailed granule neuron models reproduces the spike raster for *in vivo* firing dynamics and was similar to Figure 2. A short burst of 5 spikes at 500 Hz is passed through the MF as inputs. Inhibition (b) blocked the spikes. Network without inhibition (a) shows 1–7 spikes via granule cells (Grcs).

To understand combinatorial effects in the granular network layer and impacts of double mossy fiber bundle stimulation, combined excitation-inhibition in the network was simulated. The “spots” are maps of excitatory activity as seen in the cerebellar granular layer [8] when MF rosettes were stimulated [8]. In the model configuration (see Table 6), the center of the spot receives stronger excitatory inputs and the consecutive peripheral neurons receive weaker excitatory input, thereby expressing a center-surround configuration (see Figure 6). Both network models could reproduce the firing dynamics [8] as well as the center-surround structure (see movie, Supplementary Material available online at doi: 10.115/2012/359529). Spiking activity was reconstructed with the morphology (see Section 2.4). A single spike through the mossy fiber activates the center followed by the periphery and the Golgi-granule circuit.

Simulation of LTP and LTD induction *in vitro* and *in vivo* on the center-surround spots was modeled by varying release probabilities and intrinsic excitability. The cells in the granular layer network receive GABAergic synaptic inputs equal to the number of excitatory inputs given to the cells in the granular layer network (see Table 5). The high reproducibility indicates that the center-surround organization was a consequence of alternating transitions between burst and silent states at granule cells was not due to the temporal jitter of MFs [54].

The center-surround structures have complex transmission properties: compared to the surround, the center detects burst on a broader band and emits bursts with shorter lag, higher frequency, and longer duration [1]. Purkinje cells overlaying above these structures may be activated, at the same time, enhance inhibition around them, explaining the spot-like organization of molecular layer responses *in vivo* [8].

### 3.6. Local Field Potential and Selective Blocking of NMDA

Understanding population code through reconstructions was essentially done to suggest how encoding of spike information may happen in cerebellar cortex. Currently, encoding of population activity is explored in microcircuits via comparisons of spatiotemporal properties of simulated neural activity and with experimental measurements using multielectrode recordings [55, 56] or two-photon imaging of activity in blocks of tissue [57, 58]. Evoked responses from granular layer *in vitro* [19] have been reconstructed computationally [13, 26]. We used the “spot” to generate and test nature of local field potentials. The postsynaptic evoked LFP response varied as per input pattern and for a combination of 3 MF and 4 MF synaptic activation, spikes were generated. With 4 MF synapses active, a doublet was seen [19]. Correspondingly the responses generated N_2a_ wave and the doublet caused the N_2b_ wave. Inhibition at time = 24 ms via GABAergic synapses suppressed the spike doublet and thereby suppressed the N_2b_ wave [8] in the evoked LFP response.

Different segments of the network generated varied evoked LFP signals due to the nature of excitatory-inhibitory balance in the network reflecting a relationship different from the extracellular components of a single neuron (see Figure 9 in [13]). We assumed extracellular space in granular layer to be isopotential [26] due to close packing of granule neurons. The simulations closely followed experimental results [8], suggesting that electrotonic compactness of granule neurons contribute to the seemingly linear relationship from granule cell clusters in the granular layer extracellular space. The variations in the nature of spike with number of spiking cells could suggest that sparse coding could be preserved as suggested by Marr [4] and Albus [3].

**Figure 7:**
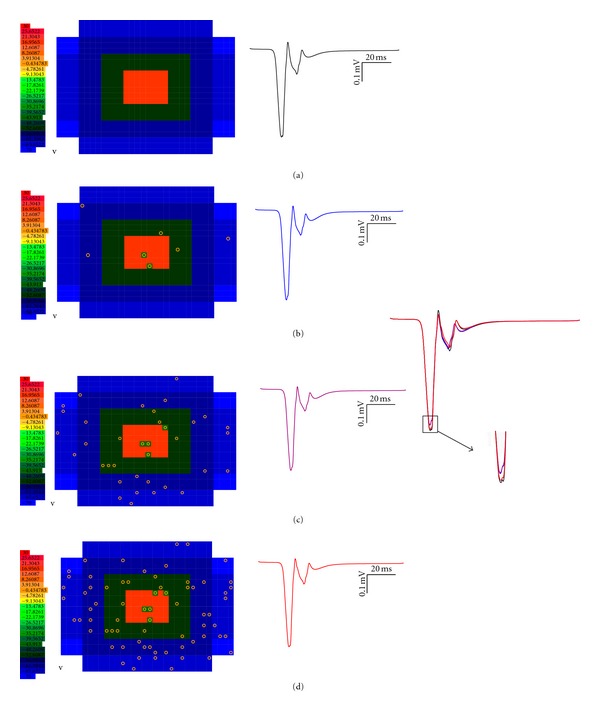
“Spot” activation and evoked LFP with selective NMDA dysfunction. Population code via evoked LFP *in vitro* response was reconstructed. Control (a) shows the clear reproducibility of N_2a_ and N_2b_ waves [18]. NR2A/NR2B knockouts show selective dysfunction of NMDA receptors. 1% (b), 5% (c), and 10% (d) cells with NMDA receptor blocked do not show much difference in population code although intracellular spiking remains altered (not shown). A small decrease in peak amplitudes was noticed. The robustness of the population code during spiking in granule cells adds to the sparse recoding theory [4] and clearly granule neurons favour their role as signal-to-noise enhancers for sensory and tactile information received via the mossy fibers (MFs).

Blocking NMDA receptors [59] in granule neurons showed reduced excitation. Selective disabling of NMDA receptors, as noticed in mice with NR2A/NR2B [59] mutations, showed decreased number of spikes which is also seen as a change in N_2a_ amplitude compared to control (Figure 7(a)). In order to predict on the nature of such mutations affecting network computation (in addition to affecting the number of spikes), we randomly disabled (1%, 5%, and 10% of total cells) NMDA receptors in the network (see circles in Figures 7(b), 7(c), and 7(d)) and reconstructed the local field response.

Disabling NMDA receptors in 1–10% of cells showed a 2.5% decrease in number of spikes in a spot (the number of spiking neurons in a 720-cell “spot” changed from 240 to 234). The network model clearly showed a “*seemingly linear*” outlook in propagating the nonlinearities of individual neurons in population code (evoked LFP, see Figures 7(a) to 7(d)). This “sense of linearity” in population code was observed also when the number of affected neurons was very low (neurons with NMDA disabled were only 1–10% of total cells). NMDA knockout mice show errors in cerebellar motor learning [59]. Plasticity changes were also reflected in the evoked LFP waves. As seen in reconstructions based on single granule neuron simulations [26], the LFP simulations on the network showed that LTP and LTD were accompanied by changes in the proportion of discharging granule cells (data not shown).

## 4. Discussion

Exploring the geometry of excitation and inhibition in cerebellar granular layer, the simulations highlight the modulatory role of inhibitory inputs on the activities of granule cells. The paper details the effects of combined excitation and combined feed-forward inhibition [8] on spiking in the granular layer. The study did not simulate feed-back inhibition coming from Golgi cell since it did not affect the modulation of 4 ms time-window that happens because of the early mossy inputs.

The simulations varying inhibition suggest that granule neurons can generate selective responses by varying synaptic strengths. The increase in spikes and modulation during plasticity indicates that the circuit is well adapted to generate enhanced responses such as in theta-burst patterns [9].

Both *in vitro* and *in vivo* simulations indicate that inhibitory input cannot completely block excitation in the network. However, it acts as a modulator that regulates the postsynaptic excitability. Both models support that burst-burst transmission modality in granule neuron and the granular layer through which high-frequency spike trains are more reliably transmitted. The consequences of transformation of spike inputs from mossy fibers to corresponding codes suggest the variable impulse response scheme indicated by previous study [10] and suggest the granular layer network also operates as an adaptive filter.

The variations of excitatory inputs (without combination of inhibition) showed differences in number of spikes and spike amplitude and did not show variations in first-spike latency [60]. The most promising outcome in variation of spikes and network spiking behavior was with the induction of LTP/LTD where both intrinsic excitability and excitatory release probabilities [21, 43] change the nature of information flow.

Simple spiking neuron models can be tuned to function as network models for accessing timing information. Spatial information in network models [19] was not seen while using spiking models. Synaptic functions in spiking models are not very reliable. Artificial models have limitations unlike biophysical models for understanding certain population activities like generation of LFP [13].

The detailed model was used as a test bench to explore the parameter space and induced plasticity. Epileptic seizure-like symptoms seen in voltage-gated sodium channel binding-related knockout mice granule neurons [7, 50] suggests that sparse and asynchronous neuronal activity can evolve into a single hypersynchronous cluster with elevated spiking rates at seizure initiation. The detailed network model suggests that LTP favors burst-burst transmission favoring high-frequency spikes. The presynaptic mechanism coexisted with postsynaptic regulation of ionic channels, which played a major role in determining the granule cell output firing frequency. Intrinsic bursting and modulatory effects of inhibition can be seen by mechanistic control of number of spikes in a granule cell.

With increased excitation, along with an increase in spikes, first-spike latency also decreased. This will also impact the local field potential and could probably explain the observations *in vitro* [18]. Both *in vitro* and *in vivo* simulations indicate that the number of spikes was dependent on the release probability of the synapses, while higher or lower intrinsic excitability caused slight change in spike amplitude.

The key role of local circuit inhibition in determining granular layer combinatorial operations was supported by several model-based predictions. Increasing active inhibitory connections saw lesser number of spikes in the network. *In vivo* bursts along mossy fibers combined with inhibitory input showed a consistent reduction of at least one spike as inhibition increased. The simulations indicated that the response of those granule cells that are intensely activated will favor with the generation of a burst, whose duration is limited by a brisk feed-forward inhibition in the Golgi cell. Inhibition controlled the number of spikes, thereby modulating spike transmission in the granular layer. The simulations suggest that erratic spikes in the mossy fibers will not be efficiently transmitted so that the burst-burst mechanism would indeed play a role in secure transmission along the mossy fiber pathway [48]. The studies also show that excitation and inhibition may consequently allow complex patterns to be processed [11].

The paper also shows population signals and effects of mechanism changes on individual neuron affecting population code generated by the network. Reconstructing extracellular properties indicated that plasticity may have similar mechanisms of burst regulation as granule cell burst initiation and may implement an adaptable delay affecting downstream activation into circuitry. The granular layer model indicates a “*seemingly linear*” tendency to propagate the nonlinearities of individual neurons via the population code even when the variations are little (affected cells 1–10% of total). The simulations suggest that a combined mechanism of NMDA blocking the After-hyperpolarization (AHP) and role of inhibition can help reconstruct transient suppression of spikes *in vitro *reported during seizures.

The studies on intensity of mossy fiber synapses and inhibitory synapses help to understand spatiotemporal operations [8] in the cerebellar granular layer. Combining granule neurons and Golgi cell, this study will help to reveal coincidence detection properties and spatial pattern separation [3]. This work is a preliminary start in modeling to understanding long sought spatiotemporal filtering predicted by the motor learning theory [61].

## 5. Conclusion

Simulations suggest how cerebellum granular layer processes spike information and how afferent information may reach cerebellar cortex and predict how spikes are processed as indicated in the sparse recoding hypothesis [4]. The role of inhibition and plasticity may help fine tune the “sparseness” of the code as indicated in Marr's theory [3, 4]. To evaluate the exact role of firing, a closer view of cells in the region of interest may be needed. The experimental testing of these predictions will require further electrophysiological and imaging investigations of granular layer activity and computational modelling of the cerebellum [1] and of the cerebro-cerebellar control loops [62, 63].

## Supplementary Material

“Spot” center surround activation in 1680 detailed biophysical network model. Note the top square (slightly larger) over the band is the Golgi cell. With a short burst through mossy fibers, the cells in the center are first activated and then the periphery. The center detects burst on a broader band and emits bursts with shorter lag, higher frequency and longer duration [1]. We also simulated the “spot” on the AdEx spiking neuron based network model. The speed in Adex model (data not shown) and detailed biophysical model are different. However, AdEx network model could reproduce the spiking patterns whereas the detailed model could reproduce both firing and ionic channel properties such as extracellular evoked potentials (see Fig. 7).

## Figures and Tables

**Table 1:** Parameter values used for AdEx spiking models.

Model	*C* (pF)	*E*_*L*_ (mV)	*V*_*T*_ (mV)	*V*_*r*_ (mV)	*b* (pA)	*g*_*L*_ (nS)	Δ*T* (mV)	*a* (nS)	*t*_*w*_ (ms)
Granule spiking model	150	−70	−50	−64	250	10	4	9	13
Golgi spiking model	350	−58	−60	−50	1460	12	7	12	7

**Table 2:** Maximal synaptic conductance values used for AMPA and GABA receptor kinetics in the simple spiking network.

Number of excitatory connections	Number of inhibitory connections	Without inhibition		With inhibition	
		Excitatory maximal conductance (nS)	Inhibitory maximal conductance (nS)	Excitatory maximal conductance (nS)	Inhibitory maximal conductance (nS)
1	4	0.14	0	0.1	0.05
2	3	0.25	0	0.11	0.1
3	2	0.256	0	0.256	0.11
4	1	0.27	0	0.3	0.18

**Table 3:** Total number of spikes in the granular layer network with biophysically detailed neuron models.

Condition	*In vitro*		*In vivo*	
	Without inhibition	With inhibition	Without inhibition	With inhibition
LTP	2736	864	4032	2304
Control	720	576	3600	2160
LTD	0	0	2016	864

Total number of spikes observed in the network under different conditions like LTP (high intrinsic excitability and higher release probability), control, and LTD (lower intrinsic excitability and lower release probability). Observe that there are no spikes *in vitro* during LTD. Golgi inhibition operates a time-window causing a significant reduction in spikes.

**Table 4:** Modulation of spiking cells *in vivo* with varying release probability in the detailed network model.

MF release probability	MF input, 5 spikes/burst		MF input, 9 spikes/burst	
	Number of spiking cells	Number of nonspiking cells	Number of spiking cells	Number of nonspiking cells
0.1	1416	264	1416	264
0.2	1416–840	840–264	1416–840	840–264
Control, 0.4	1416–840	840–264	1416–840	840–264
0.5–0.8	1416–840	840–264	1416–840	840–264

**Table 5:** Effect of inhibition on number of spikes in the detailed network model.

Number of inhibitory fiber synapses	*In vitro*				*In vivo* (short burst)				*In vivo* (long burst)			
	4 MF	3 MF	2 MF	1 MF	4 MF	3 MF	2 MF	1 MF	4 MF	3 MF	2 MF	1 MF
0	2	1	0	0	7	5	3	0	8	6	3	0
1	2	1	0	0	6	4	2	0	7	5	2	0
2	1	1	0	0	5	3	0	0	6	4	1	0
3	1	1	0	0	4	2	0	0	5	3	0	0
4	1	1	0	0	3	1	0	0	4	2	0	0

The table shows the effect of inhibitory synapses on the spikes under different conditions (*in vitro*, *in vivo* (short burst); *in vivo* (long burst)). Under each condition, first row denotes increasing number (1–4) of excitatory synapses from right to left while increasing number of inhibitory synapses (0–4 in first column). With increase in number of inhibitory synapses (0–4), the number of spikes observed in the cells decreases. A gradual decrease in number of spikes with increased inhibition can be observed.

**Table 6:** Center-surround pattern and spiking cells of detailed granular layer network.

Number of cells	Number of active MF synapses	Number of spikes	
		Network without inhibition	Network with inhibition
144	4	7 spikes/burst	6 spikes/burst
432	3	5 spikes/burst	3 spikes/burst
144	2	2 spikes/burst	EPSP
432	1	EPSP	EPSP

^a^Cells with 4 excitatory inputs produced 7 spikes/burst when inhibitory synapse was switched off and produced 5 spikes/burst when it was switched on.
